# Retinoic acid‐loading of the major birch pollen allergen Bet v 1 may improve specific allergen immunotherapy: In silico, in vitro and in vivo data in BALB/c mice

**DOI:** 10.1111/all.14259

**Published:** 2020-04-16

**Authors:** Karin Hufnagl, Sheriene Moussa Afify, Nina Braun, Stefanie Wagner, Michael Wallner, Michael Hauser, Markus Wiederstein, Gabriele Gadermaier, Sabrina Wildner, Frank A. Redegeld, Bart R. Blokhuis, Gerlinde Hofstetter, Isabella Pali‐Schöll, Franziska Roth‐Walter, Luis F. Pacios, Erika Jensen‐Jarolim

**Affiliations:** ^1^ The interuniversity Messerli Research Institute of the University of Veterinary Medicine Medical University of Vienna and University of Vienna Vienna Austria; ^2^ Laboratory Medicine and Immunology Department Faculty of Medicine Menoufia University Shebin El‐Kom Egypt; ^3^ Department of Biosciences University of Salzburg Salzburg Austria; ^4^ Protein Bioinformatics Research Group Department of Biosciences University of Salzburg Salzburg Austria; ^5^ Division of Pharmacology Utrecht Institute for Pharmaceutical Sciences Faculty of Science Utrecht University Utrecht The Netherlands; ^6^ Centre for Plant Biotechnology and Genomics CBGP (UPM‐INIA) and Department of Biotechnology‐Plant Biology Universidad Politécnica de Madrid Madrid Spain; ^7^ Institute of Pathophysiology and Allergy Research Center of Pathophysiology, Infectiology and Immunology Medical University Vienna Vienna Austria; ^8^ Biomedical International R+D GmbH Vienna Austria

**Keywords:** Bet v 1, birch pollen allergy, immunotherapy, mouse model, retinoic acid

**Abbreviations**
ANS: 1‐anilino‐8‐naphthalene sulphonate
BCA: bicinchoninic acid
BP: birch pollen
DMSO: dimethyl sulphoxide
E_af__f_: affinity energy
FCS: foetal calf serum
IL: interleukin
K_D_: binding constant
kDa: kilodalton
OD: optical density
PBMCs: peripheral blood mononuclear cells
PDB: protein data bank
RA: retinoic acid
RBL: rat basophilic leukaemia
RFUs: relative fluorescence units

To the Editor,

More than twenty different isoforms of Bet v 1, the major birch pollen allergen, have been identified, sharing an amino acid sequence identity of 95% and an almost identical tertiary structure.^1^ Despite their structural similarities, the isoforms display remarkable different immunogenic properties and IgE‐binding capacities.^2^

Bet v 1 isoforms were recently shown to differ in ligand binding concerning small hydrophobic plant mediators, which could relate to the diverging immunogenic and allergenic properties of the Bet v 1 isoforms.^3^ We found that Bet v 1a (Bet v 1.0101), structurally comparable to human lipocalin‐2, is able to bind iron via catechol‐based siderophores in its internal cavity.^4^ When incubated with human immune cells, only the unloaded *apo‐*Bet v 1 molecule caused Th2 cells to secrete IL‐13.^4^ Our search for other ligands able to induce immunomodulation, supported by data from literature and in silico docking calculations, led us to the major vitamin A metabolite retinoic acid (RA). RA has not only intrinsic immunomodulatory properties,^5^ it is also able to abrogate the Th2 immunogenicity of the major milk allergen Bos d 5 when in *holo*‐form.^6^ In the present study, we hence concentrated on the ability of Bet v 1a and of the hypoallergenic isoform Bet v 1d (Bet v1.0102) to bind RA in their internal cavity and the subsequent effects on their allergenic potential.

First, we investigated whether RA at all is able to bind into the cavity of Bet v 1 utilizing in silico docking analysis and an in vitro ANS competition assay (Figure 1A, D). In silico calculations, using the crystal structure of the Bet v 1‐naringenin complex (PBD entry 4A87) for Bet v 1a (Figure 1A) and a homology model template based on PDB entry 4MNS for Bet v 1d (Figure 1D), revealed an identical affinity energy of −8.7 kcal/mol for both isoforms, corresponding to a dissociation constant of 0.364 µmol/L. The close‐up view of the RA‐binding site in the intramolecular cavity of Bet v 1a (Figure 1A) and Bet v 1d (Figure 1D) shows a hydrogen bond between oxygen atoms of RA and Asp27. The second hydrogen bond relates to residue Tyr81 and residue Lys54 for Bet v 1a and Bet v 1d, respectively. Our in silico findings were corroborated by an in vitro ANS competition assay showing that RA dose‐dependently displaced ANS from both Bet v 1a (Figure 1 A) as well as Bet v 1d (Figure 1D), which indicates that principally both Bet v 1 isoforms are able to bind RA in their hydrophobic cavity.

**FIGURE 1:**
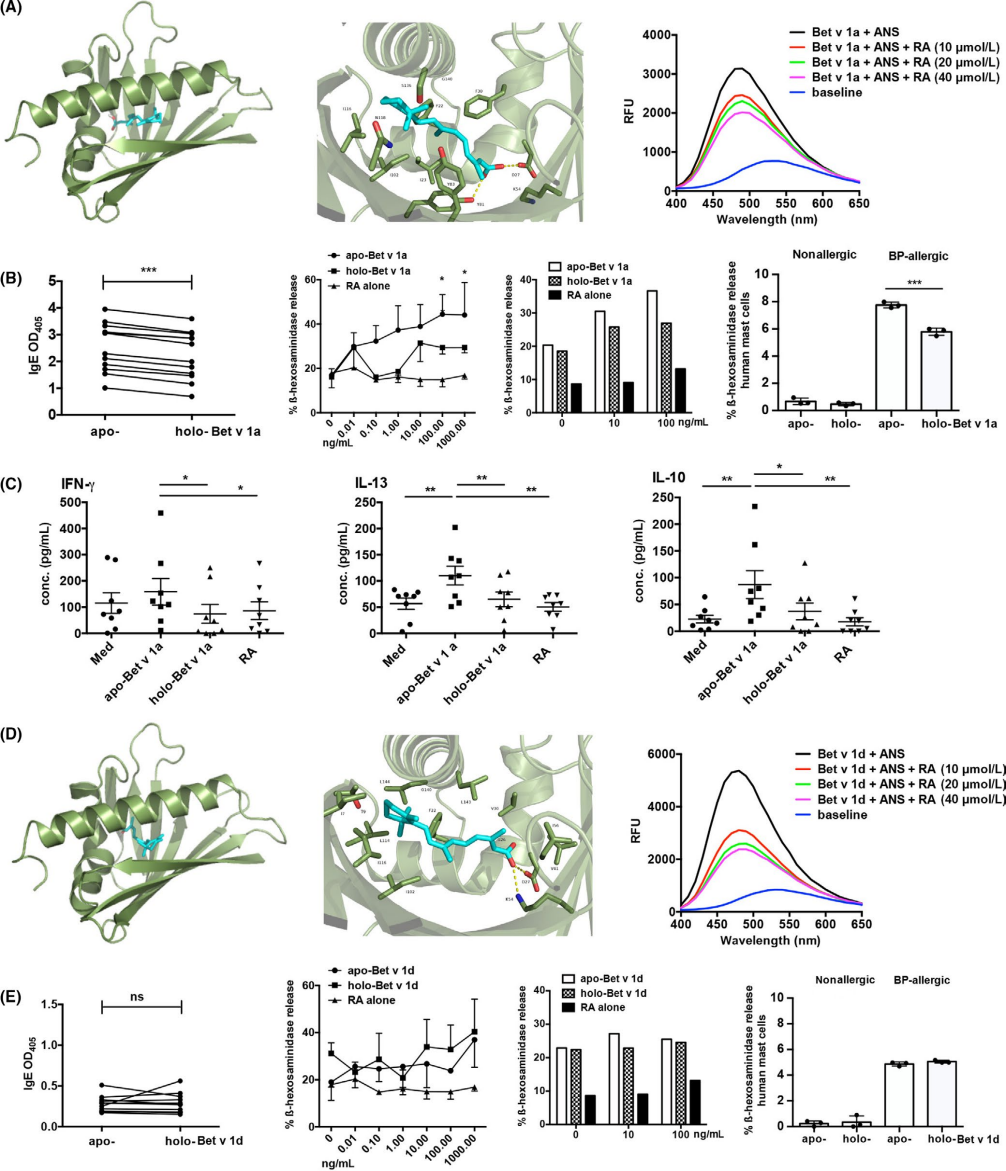
Bet v 1 can be loaded with RA, leading to reduced IgE binding, IgE cross‐linking and cytokine production in vitro. In silico docking analysis, close‐up view of the cavity and ANS competition assay of (A) Bet v 1a and of (D) Bet v 1d with ligand RA (sticks in turquoise); IgE levels in sera of 12 BP‐allergic individuals (ELISA, paired samples *t* test) and ß‐hexosaminidase release from humanized RBL cells sensitized either with serum IgE from three BP‐allergics or with a serum pool from ten BP‐allergic individuals against (B) *apo‐* or *holo‐*Bet v 1a and (E) *apo‐* or *holo*‐Bet v 1d (2 way ANOVA followed by Bonferroni multiple comparison test); (B, 4th graph) Bet v 1a‐induced and (E, 4th graph) Bet v 1d‐induced ß‐hexosaminidase release from human mast cells sensitized with serum pools from nonallergic and BP‐allergic donors (ANOVA followed by Tukey's multiple comparison test); (C) IFN‐γ, IL‐13 and IL‐10 levels (pg/mL) in PBMCs from eight BP‐allergic donors stimulated in vitro with *apo‐* or *holo*‐Bet v 1a (ANOVA followed by Newman‐Keuls multiple comparison test); means ± SEM, *P < .05, **P < .01, and ***P < .001; RFU, relative fluorescence units; OD, optical density; ns, nonsignificant

Next, we investigated whether RA binding has an influence on the IgE‐binding capacity of the Bet v 1 isoforms. In ELISA *holo‐*Bet v 1a (+ ligand RA) showed significantly reduced binding of serum IgE compared to the unloaded *apo*‐Bet v 1a (Figure 1B). Not only the IgE binding but also the IgE cross‐linking abilities of Bet v 1a were affected by RA, as *holo‐*Bet v 1a (+ ligand RA) induced significantly less ß‐hexosaminidase mediator release from RBL‐SX38 cells (Figure 1B). Similarly, mediator release in primary human mast cells, presensitized with IgE from sera of BP‐allergic donors, was significantly reduced by *holo‐*Bet v 1a treatment (Figure 1B). In contrast, both *apo*‐ and *holo*‐Bet v 1d displayed lower IgE‐binding capacity (Figure 1E), and we found no significant differences in mediator release independent whether the unloaded *apo‐* or the RA‐loaded *holo‐*form of Bet v 1d was used in the RBL assay or in primary human mast cells (Figure 1E). Subsequent in silico analysis revealed that the IgE‐specific effects may be due to epitope masking by ligand RA, as RA binding could interfere with two described IgE‐binding B‐cell epitope regions in the Bet v 1 structure (Figure S1A). Preliminary in vitro data showed that this effect reached significance with RA only, and not with control ligands epinephrine and catechol (Figure S2).

In this context, we also found that protein residues Glu142 and Leu144, representing important T‐cell epitope residues of Bet v 1a within the major T‐cell epitope, are in favourable position to interact with RA (Figure S1B). Thus, we hypothesized that also cellular immune responses could be influenced by ligand RA binding. Therefore, we incubated PBMCs from BP‐allergic donors with unloaded or RA‐loaded Bet v 1a and measured the cytokines released into supernatants. PBMCs from BP‐allergic donors produced significantly less IFN‐**γ**, IL‐13 and IL‐10 when stimulated with RA‐loaded hyperallergen, *holo*‐Bet v 1a, than with unloaded *apo*‐Bet v 1a (Figure 1C). Even if this was not due to altered endolysosomal stability of Bet v 1a due to RA binding (Figure S3), it seemed so far like RA‐loading transforms the hyperallergen Bet v 1a to a hypoallergen with improved tolerogenic capacity of potential implications for allergen immunotherapy.

We aimed to challenge this hypothesis in a therapeutic mouse model of birch pollen allergy (Figure S4). Mice were first made allergic against the major birch pollen allergen Bet v 1a and subsequently treated intranasally with either the *apo*‐Bet v 1a, or with the RA‐loaded *holo*‐Bet v 1a, or as a control with RA alone. The therapeutic application of *holo*‐Bet v 1a significantly prevented body temperature drop and anaphylactic symptoms upon a specific allergen challenge compared to control mice (Figure 2A). Mice treated with *apo*‐Bet v 1a displayed anaphylactic symptom levels comparable to the control group (Figure 2A). This effect was recorded and visualized in the noninvasive anaphylaxis imaging cage,^7^ where more constant body temperature and unchanged physical activity were evident in the *holo*‐Bet v 1a treated group (Figure 2B). The alleviated allergic symptoms in *holo‐*Bet v 1a treated mice were accompanied by significantly enhanced allergen‐specific IgG2a, IgG2b and IgA serum levels, while *apo‐*Bet v 1a treated mice showed no significant changes compared to control mice (Figure 2C). Allergen‐specific IgE levels were not affected by any treatment (Figure 2C). Similar alterations of allergen‐specific IgE and IgG responses can be seen during human allergen‐specific immunotherapy.^8^ Systemic immune response was analysed by cytokine expression in supernatants of splenocytes in vitro stimulated with Bet v 1a (Figure 2D). Significant changes were found in spleen cells from *holo‐*Bet v 1a treated mice, showing significantly enhanced IL‐10 levels compared to medium control values (Figure 2D). Th1 (IFN‐γ) and Th2 (IL‐13) responses (Figure 2D) as well as the percentage of CD4 + CD25+ splenic T cells (Figure S5) exhibited no significant changes after *apo*‐ or *holo*‐Bet v 1a treatment.

**FIGURE 2:**
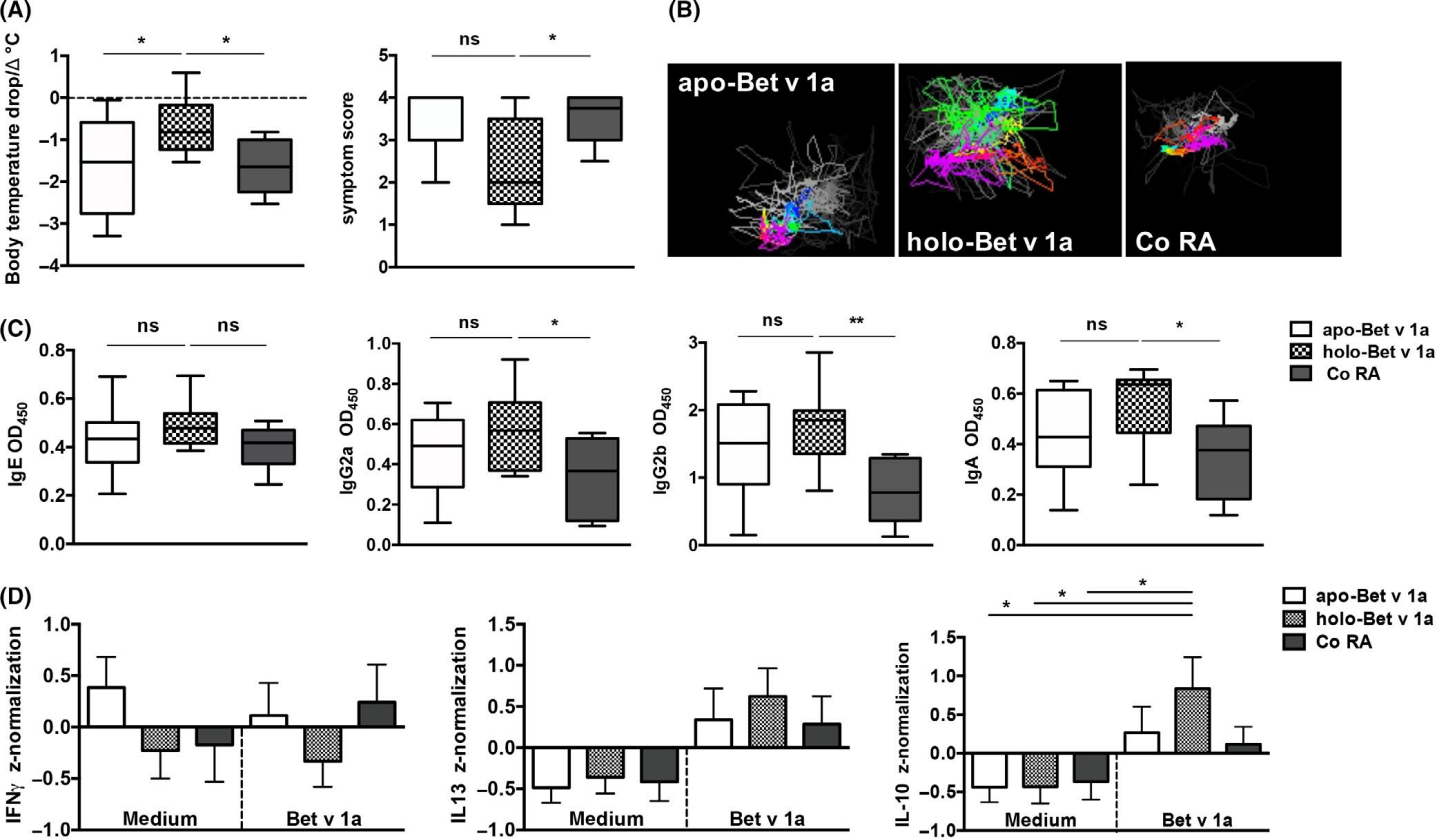
RA‐loading of Bet v 1 improves specific allergen immunotherapy in a mouse model. After sensitization to Bet v 1a, mice were therapeutically treated intranasal with *apo‐*Bet v 1a*, holo‐*Bet v 1a or RA alone, and then subjected to a specific allergen challenge, again with Bet v 1a (Figure S3); (A) body temperature drop and symptom score 20 min after i.p. Bet v 1a‐challenge (ANOVA followed by Tukey's multiple comparison test); (B) representative images of body temperature (blue to red indicates low to high temperature) and movements (lines) recorded by the imaging cage; (C) Bet v 1a‐specific serum IgE, IgG2a, IgG2b and IgA levels from sensitized mice treated with *apo‐*Bet v 1a*, holo‐*Bet v 1a or RA alone (ANOVA followed by Tukey's multiple comparison test). (D) IFN‐γ, IL‐13 and IL‐10 production in mouse splenocytes after in vitro stimulation with medium or Bet v 1a in respective treatment groups. Z‐normalization of pg/mL for each cytokine was performed as described in material and methods (ANOVA followed by Tukey's multiple comparison test). Graphs show pooled results from two independent experiments with total n = 11 mice per group in mean ± SEM; *P < .05; **P < .01; OD, optical density; ns, nonsignificant

Thus, we were able to demonstrate for the first time that loading of RA into the hydrophobic pocket of Bet v 1a reduces its IgE‐binding and cross‐linking abilities to human primary mast cells in vitro, and alleviates allergic symptoms in vivo. Carotenoids and fatty acids naturally occur in pollen and exhibit anti‐oxidant properties.^9^ The high binding affinity in the nanomolar range of RA into the pocket of Bet v 1 suggests that such molecules might naturally synergize with the birch pollen allergen. When during environmental stress the pathogenesis‐related Bet v 1 molecule gets overexpressed, a resulting predominance of insufficiently loaded *apo*‐allergens may contribute to the allergy epidemic.

While the underlying mechanisms of immunomodulation by RA‐loading are still not fully understood, we propose that ligand binding can be decisive for the development of tolerance and as a future perspective could be helpful to improve immunotherapeutic approaches in birch pollen allergy.

## CONFLICT OF INTEREST

Dr Hufnagl, Dr Afify, Nina Braun M.Sc., Dr Hauser, Dr Wiederstein, Dr Wildner, Dr Redegeld, B. Blokhuis, G. Hofstetter, Dr Pali‐Schöll and S. Wagner M. Sc. have nothing to disclose. Dr Wallner reports a FWF grant P 23417. Dr Gadermaier reports personal fees from Bencard and personal fees from Compare database, outside the submitted work. Dr Roth‐Walter reports grants from Bencard grant award 2018 and personal fees as a research consultant for Biomedical International. R + D GmbH, outside the submitted work; in addition, Dr Roth‐Walter has a patent EP2894478, owned by Biomedical Int. R + D issued. Dr Pacios has a patent EP2894478 issued and a patent US15111162 issued. Dr Jensen‐Jarolim reports other from Biomedical International R + D GmbH, Austria, grants from Bencard Allergie GmbH, Germany, during the conduct of the study; in addition, Dr Jensen‐Jarolim, Dr Roth‐Walter and Dr Pacios have a patent EP 2894478 A1, US20160334418 and WO2015104270A1 issued.

## Funding information

This work was supported by the SFB F4606‐B28 grant of the Austrian Science Fund FWF.

## Supporting information

Supplementary Material

Fig S1

Fig S2

Fig S3

Fig S4

Fig S5
