# Supplementary material for: Retinoic acid‐loading of the major birch pollen allergen Bet v 1 may improve specific allergen immunotherapy: In silico, in vitro and in vivo data in BALB/c mice
Source: Allergy. 2020 Apr 16;75(8):2073–7. doi: 10.1111/all.14259 (PMC7522679; doi:10.1111/all.14259)
Supplement: Supplementary file 1 — Supplementary Material [file ALL-75-2073-s001.docx]

**Supplementary Information**

**Retinoic acid-loading of the major birch pollen allergen Bet v 1 may improve specific allergen immunotherapy: *in silico,* *in vitro* and *in vivo* data in BALB/c mice**

Karin Hufnagl, PhD^1^, Sheriene Afify, MD, M.Sc. ^1,2^, Nina Braun, M.Sc.^1^, Stefanie Wagner, M.Sc.^1^, Michael Wallner, PhD^3^, Michael Hauser, PhD^3^, Markus Wiederstein, PhD^4^, Gabriele Gadermaier, PhD^3^, Sabrina Wildner, PhD^3^, Frank A. Redegeld, PhD^5^, Bart R. Blokhuis, M.Sc.^5^, Gerlinde Hofstetter, M.Sc.^1^, Isabella Pali-Schöll^1^, PhD, Franziska Roth-Walter, PhD^1^, Luis F. Pacios, PhD^6^, Erika Jensen-Jarolim, MD^1,7,8^

**Material and Methods**

***Materials***

All *trans*-retinoic acid and deferoxamine mesylate were purchased from Sigma (Sigma Aldrich, Steinheim, Germany). Ficoll-Paque PLUS was from GE Healthcare (Uppsala, Sweden). Human and mouse ELISA kits for IL10, IL13 and IFNy were obtained from Invitrogen/Thermo Fisher Scientific (Waltham, MA, USA). Recombinant Bet v 1d was kindly donated by M. Wallner from the University of Salzburg^S1^.

***In silico docking analysis***

*Structures: Bet v 1a*: The geometry of Bet v 1a was taken from the high-resolution (1.24 Å) crystal structure of its complex with naringenin (protein data bank (PDB) entry 4A87)^3^.

*Bet v 1d*: As no experimental structure is available for this isoform, Bet v 1d was modelled using MODELLER^S2^ with a high-resolution (1.2 Å) crystal structure of Bet v 1a as template (PDB entry 4MNS)^S3^. The resulting model was evaluated with ProSA^S4^ and MAESTRO^S5^. As expected from the high sequence similarity between target and template (96% identities), model evaluation scores were in the range of experimentally determined Bet v 1 structures and indicated high model quality.

*Docking calculations*: AutoDock Vina was used to obtain the geometries of the ligand RA bound into the hydrophobic pocket of Bet v 1a and its isoform through docking calculations^S6^. The geometries selected were those having the lowest affinity energies (E_aff_) (best docking solutions) that were then used to estimate the dissociation constant K_D_ for the protein-ligand complexes under the assumption E_aff_ ~ ΔG with K_D_ = exp(-ΔG/RT) at *T* = 298.15 K^4^.

Figures 1 A and D were prepared and rendered with PyMOL (The PyMOL Molecular Graphics System, Version 2.1 Schrödinger, LLC 2015). Close-up views of the RA binding site were defined by a 3.7 Å radius around RA atoms in the internal cavity of Bet v 1a and Bet v 1d with hydrogen bonds shown as broken lines.

***Recombinant Bet v 1a***

Bet v 1 was produced as described ^S7^. In short, a codon-optimized synthetic gene of Bet v 1.0101 were obtained from Eurofins MWG Operon (Ebersberg, Germany) and cloned into the expression vector pET-28a(+) (Merck Millipore, Darmstadt, Germany). The proteins were expressed in E. coli BL21[DE3] in LB medium at 37 °C after induction with 1 mM isopropyl--D-thiogalactopyranoside. Bet v 1 was purified by a combination of hydrophobic interaction and ion exchange chromatography. SDS-PAGE, MALDI-TOF MS (Bruker Ultraflex II, Bruker Daltonics, Billerica, MA, USA), and circular dichroism (CD) spectroscopy were used to verify protein purity and identity, mass, and secondary structure. Measurement of endotoxin content was done by Hyglos EndozymeII Kit (Bernried am Starnberger See, GER) and total protein content by BCA assay according to the manufacturer’s instructions (Pierce BCA Protein Assay Kit, Thermo Scientific, Rockford, IL, USA).

***Generation of apo- and holo-Bet v 1a/Bet v 1d***

Bet v 1a (1 mg/ml) and Bet v 1d (0.8 mg/ml) were dialyzed three-times against 10 µM deferoxamine mesylate salt. Further dialyzation against deionized water was performed to receive *apo-*Bet v 1 isoforms. For ELISA and RBL degranulation assay *apo-*Bet v 1 isoforms were incubated over night with a 5-fold molar excess of ligand RA dissolved in dimethyl sulfoxide (DMSO)^6^. Control experiments included 5-fold molar excess of ligand catechol (Sigma Aldrich) and epinephrine (Sigma Aldrich) in DMSO or 0.5 M HCl respectively. For *in vitro* experiments with human PBMCs 2.5 µM *apo-*Bet v 1a was pre-incubated with 1 µM RA. Control cells received DMSO and RA in the respective concentrations^6^. For mice experiments *apo-*Bet v 1a was pre-incubated with a 2-fold molar excess of ligand RA dissolved in ethanol (Figure S4).

***ANS competition assay***

Bet v 1a or Bet v 1d (10 µM final concentration) were incubated with RA in 96-well UV-Star plates (Thermo Fisher Scientific, Waltham, MA, USA) in different ligand-to-protein molar ratios (1:1 – 1:4; 10, 20 or 40 µM) overnight at 4°C under light protection. Prior to measurement 5 µL ANS solution (50 µM final concentration) was added and the mixtures were incubated for another hour at room temperature. Controls consisted of RA, Bet v 1a, Bet v 1d and ANS alone as well as Bet v 1a or Bet v 1d with ANS. Absorbance spectra were recorded at λexc = 350 nm and λemi = 300 to 800 nm in 10 nm steps. Read-outs for both assays were performed on a TECAN Plate Reader Infinite M200 PRO^6^.

**Endolysosomal Degradation Assay**

The proteolytic stability to endolysosomal proteases was studied using the endolysosomal degradation assay. The endolysosomal fraction was isolated from murine JAWS II dendritic cells by differential centrifugation as previously described^S8^. Five µg of the protein were incubated with 7.5 µg of isolated microsomal proteins (37.5 µg) in a final volume of 20 µL containing 100 mM citrate buffer pH 4.8 and 2 mM dithiothreitol. The proteolytic digestion at 37 °C was monitored up to 72 h. Reactions were terminated by denaturation at 95 °C for 5 min at the time points of 0, 0.5, 1, 3, 8,16, 24, 48 and 72h, and sodium dodecyl sulfate polyacrylamide gel electrophoresis (SDS-PAGE) was performed to monitor the process.

***Patients and sera***

Experiments with blood samples from BP-allergic patients were approved by the institutional ethics committee of the Medical University of Vienna and conducted in accordance with the Helsinki Declaration of 1975 (EK number 2007/2016). All subjects gave their full written informed consent.

***Allergen-specific IgE ELISA***

For detection of Bet v 1-specific IgE in sera of BP-allergic donors, duplicate wells of MaxiSorp 96 well flat-bottom plates (Nunc, Rochester, NY, USA) were coated simultaneously with 100 µl/well of *apo-*Bet v 1a, *apo-*Bet v 1d, *holo-*Bet v 1a or *holo-*Bet v 1d diluted at 2 µg/ml in coating buffer and incubated overnight at 4°C. After 2 h blocking at room temperature with 200 µl Tris buffered saline (TBS) + 0.05 % Tween 20 (TBS-T) + 1% BSA, wells were incubated with 100 µl of human serum diluted 1:10 in TBS-T overnight at 4°C. Horseradish peroxidase conjugated (HRP) goat anti-human IgE antibody (BD Pharmingen) diluted at 1:1000 in TBS-T was used as secondary antibody. ABTS substrate activated by H_2_O_2_ (100 µl/well; Sigma) was added to detect bound HRP conjugated antibodies. The optical density was measured at 405 nm using an Infinite M200Pro microplate reader (Tecan, Austria).

***RBL degranulation assay***

Human FcεRI-expressing rat basophil cells (RBL-SX38) (2×10^4^/well in 96 well plates) were sensitized overnight with human serum of BP-allergic donors, 1:10 diluted in Tyrode’s buffer. Sensitized cells were stimulated simultaneously with varying concentrations (0.01 - 1 ng/ml) of *apo-* or *holo-*Bet v 1a and *apo-* or *holo*-Bet v 1d in Tyrode’s buffer for 1 h at 37°C. Degranulation was assessed by measurement of released ß-hexosaminidase in the supernatant and of unreleased enzyme in the respective cell lysate. The presented results were calculated as percentage release of total ß-hexosaminidase content.

***Generation of primary human mast cells and degranulation assay***

CD34+ derived human MC were generated from surplus autologous stem cell concentrates as previously described^S9^. Briefly, frozen stem cell concentrates were rapidly thawed at 37°C under sterile conditions and poured in a large cell culture flask (Greiner). 20% human serum albumin clinical solution (HSA) (Sanquin), 6% hydroxyethyl starch clinical solution (Braun), and RMPI containing 10 U/ml Heparin (LEO pharma) were then added slowly and consecutively to the cell concentrate. Cells were then filtered through a cell dissociation sieve (Sigma) and incubated with DNAse (200 U.I./ml, Roche) for 15min. After washing, cells were re-suspended in PBS containing 4% HSA and incubated with Fc-Block (Miltenyi) for 15min, CD34+ positive selection cocktail (StemCell) for 15min and nanoparticles for 10min. Subsequently, CD34+ cells were sorted with an EasySep R Magnet (StemCell) according to the manufacturer’s protocol. Finally, sorted cells were re-suspended in serum-free expansion medium (SFEM) (StemCell) supplemented with human LDL (50μg/ml, StemCell). On day 1, human recombinant IL-3 (100 ng/ml, Biolegend), and SCF (100 ng/ml, Miltenyi) were added. Every three to four days, IL-3 and SCF were added to a final concertation of 20 ng/ml. At the end of the second week, MC were maintained under 20 ng/ml SCF with the withdrawal of IL-3. After 4 weeks, the cells were cultured in Iscove’s modified Dulbecco’s medium/ 0.5% BSA with human IL-6 (50 ng/mL, Peprotech, Rocky Hill, NJ), and 3% supernatant of Chinese hamster ovary transfectants secreting murine stem cell factor (a gift from Dr P. Dubreuil, Marseille, France). The mature MCs were identified by flow cytometry based on positive staining for CD117 (eBioscience) and Fc**ε**RIa (eBioscience) using BD FACSCanto II (approximately 90%).

Degranulation assay was performed according to protocols from RBL degranulation assay (see section “RBL degranulation assay” in the material and methods section) using serum pools from eight non-allergic and eight BP-allergic donors, followed by incubation with *apo-* or *holo-*Bet v 1a and *apo-* or *holo-*Bet v 1 d (5 nM). Degranulation was assessed by measurement of released ß-hexosaminidase in the supernatant and of unreleased enzyme in the respective cell lysate. The presented results were calculated as percentage release of total ß-hexosaminidase content. The release from unstimulated controls was 0.041 %, from positive controls with anti-human IgE 35 % and with ionomycin 94 %.

***Isolation and stimulation of PBMCs***

Heparin-treated blood (15 mL) was mixed with equal volumes of PBS containing 2% FCS before applying 10 mL Ficoll-Paque PLUS and centrifuged at 400 g for 30 minutes without brake. After density gradient separation, the lymphocyte fraction was isolated and transferred to a fresh tube. Cells were washed twice with 0.9% sodium chloride solution. Subsequently, cells were diluted to a concentration of 1×10^6^ cells/mL in RPMI medium containing 10% FCS, 2 mM L-glutamine and 1% penicillin/streptomycin. Based on previous titration experiments^4,5^ isolated PBMCs (0.5×10^6^/well) were incubated with *apo-*Bet v 1a (2.5 µM), *holo*-Bet v 1a (*apo*-Bet v 1a (2.5 µM) pre-incubated with 1 µM RA) and as controls DMSO (Med Co; 0.1% v/v) or RA (1 µM) alone. The cells were incubated for 6 days at 37°C. After 6 days supernatants were collected and stored at -80°C until further analysis.

***Determination of cytokines in supernatants of human PBMCs***

IFNy, IL-10, and IL-13 were detected with commercially available kits according to the manufacturer’s protocol (Invitrogen/Thermo Fisher Scientific). IFNy, IL-10 and IL-13 have a reported sensitivity of 4 pg/mL.

***Animals***

Female inbred 7-week-old BALB/c mice were obtained from Charles River Laboratories (Sulzfeld, Germany). Mice were kept under conventional housing and treated according to European Union rules of animal care with the permission of the Austrian Ministry of Science (BMWF-66.009/0133-WF/V/3b/2016). The combined results from two independent experiments (experiment 1: n = 4/group; experiment 2: n = 7/group; in total n = 11/group) are shown.

***Allergic sensitization and therapeutic intranasal treatment of mice***

Sensitization of mice was performed by 3 intraperitoneal (i.p.) injections of 1 µg Bet v 1a adsorbed to aluminium hydroxide (Al[OH]_3_; Serva, Heidelberg, Germany) at 14-day intervals (Figure E2). Therapeutic intranasal treatment was started 10 days after the last i.p. injection. Mice received 40 µg *apo*-Bet v 1a (*apo*-Bet v 1a group), 40 µg *apo*-Bet v 1a pre-incubated with RA (*holo*-Bet v 1a group), or as control RA alone (Co RA group) for 3 times in 6-days intervals. One week after the last intranasal treatment mice were challenged intraperitoneally with 30 µg Bet v 1a in PBS. Animals were monitored over 20 minutes for body temperature and movements using an Imaging system (Biomedical Int., Austria)^7^ and scoring of systemic anaphylaxis was done as previously described^S10^. Subsequently mice were euthanized by gradual introduction of CO_2_ and blood was collected by cardiac puncture ^S11,S12^.

***Detection of allergen-specific antibody levels in mouse sera***

Bet v 1-specific IgG2a, IgG2b, IgA and IgE were measured by ELISA. In brief, Bet v 1 (5 μg/well) was coated, blocked with 1% BSA in PBS, and incubated with diluted sera (1: 100 for IgG2a, IgG2b, IgA and 1:10 for IgE) overnight. Detection was performed by monoclonal rat anti-mouse IgG2a (clone R19-15), IgG2b (clone R12-3), IgA (clone c10-1), or IgE (clone R35-72) followed by polyclonal peroxidase-labeled goat anti-rat IgG (GE Healthcare). All primary antibodies were from BD Pharmingen. Tetramethylbenzidine (BD Biosciences) was used as substrate. The reaction was stopped with 1.8 M sulfuric acid and detected at 450 nm.

***Determination of cytokine production in mouse splenocytes***

Single-cell suspensions of splenocytes were plated at a density of 5 × 10^6^ cells/ml in 48-well culture plates (Thermo Scientific) and cultured with 10 μg/ml Bet v 1a, 2.5 μg/ml concanavalin A (Sigma), or medium alone for 72 hours. Cytokines IFNy, IL-10, and IL-13 of cultured supernatants were measured by ELISA (Invitrogen/Thermo Fisher Scientific) according to the manufacturer’s instructions. For the normalization of the geometric mean of cytokine data (pg/ml) the following formula was used:$z-normalisation= \frac{\left( x-\bar{x} \right)}{S}$, where $\bar{x}$ is the mean of the pg/ml values of the population and $S$ is the standard deviation of the pg/ml values of the population^S13^.

***Flow cytometric analyses of mouse splenocytes***

Single-cell suspensions of murine splenocytes (0.5 million cells) were stained for CD4+CD25+ activated/regulatory T cells using anti-CD4 FITC (clone RM4-5; eBioscience) and anti-CD25 APC (clone PC61.5; eBioscience) antibodies according to the manufacturer's instructions. Cells were acquired by flow cytometry (BD Bioscience Canto II). Gating on the living population and exclusion of doublets was done before gating for CD4+ CD25+ cells. Acquired cells were analysed using the FACSDiva Software 6.0 ^S12^.

***Statistical Analysis***

Statistical analyses when comparing more than two groups were calculated by ANOVA following Tukeys, Newman-Keuls or Bonferroni Multiple Comparison test using GraphPad Prism 6 software (GraphPad, San Diego, CA, USA). Differences between two groups were analyzed using unpaired t-test. Data are shown as mean +/- SEM. A value of p < 0.05 was considered significant.

**References**

1. Wallner M, Himly M, Neubauer A, et al. The influence of recombinant production on the immunological behaviour of birch pollen isoallergens. *PLoS One* 2009;4(12):e8457
2. Sali A, Blundell TL. [Comparative protein modelling by satisfaction of spatial restraints.](https://www.ncbi.nlm.nih.gov/pubmed/8254673) *J Mol Biol* 1993;234(3):779-815.
3. Nony E, Bouley J, Le Mignon M, Lemoine P, Jain K, Horiot S. [Development and evaluation of a sublingual tablet based on recombinant Bet v 1 in birch pollen-allergic patients.](https://www.ncbi.nlm.nih.gov/pubmed/25846209) *Allergy* 2015 (7):795-804.
4. Sippl MJ. [Recognition of errors in three-dimensional structures of proteins.](https://www.ncbi.nlm.nih.gov/pubmed/8108378) *Proteins* 1993;17(4):355-62.
5. Laimer J, Hofer H, Fritz M, Wegenkittl S, Lackner P. MAESTRO--multi agent stability prediction upon point mutations. [*BMC Bioinformatic*](https://www.ncbi.nlm.nih.gov/pubmed/?term=laimer+2015+maestro)s 2015;16:116.
6. Trott O, Olson AJ. [AutoDock Vina: improving the speed and accuracy of docking with a new scoring function, efficient optimization, and multithreading.](https://www.ncbi.nlm.nih.gov/pubmed/19499576) *J Comput Chem* 2010 ;31(2):455-61.
7. Guhsl EE, Hofstetter G, Hemmer W, et al. [Vig r 6, the cytokinin-specific binding protein from mung bean (Vigna radiata) sprouts, cross-reacts with Bet v 1-related allergens and binds IgE from birch pollen allergic patients' sera.](https://www.ncbi.nlm.nih.gov/pubmed/23996905) *Mol Nutr Food Res* 2014;58(3):625-34.
8. Wildner S, Elsässer B, Stemeseder T, et al. [Endolysosomal Degradation of Allergenic Ole e 1-Like Proteins: Analysis of Proteolytic Cleavage Sites Revealing T Cell Epitope-Containing Peptides.](https://www.ncbi.nlm.nih.gov/pubmed/28812992) *Internat J Mol Sci* 2017;18(8): E1780
9. Yu Y, Blokhuis BRJ, Diks MAP, Keshavarzian A, Garssen J, Redegeld FA. [Functional Inhibitory Siglec-6 Is Upregulated in Human Colorectal Cancer-Associated Mast Cells.](https://www.ncbi.nlm.nih.gov/pubmed/30294327) *Front Immunol*2018;9:2138.
10. Roth-Walter F, Berin MC, Arnaboldi P, et al. Pasteurization of milk proteins promotes allergic sensitization by enhancing uptake through Peyer’s patches. *Allergy* 2008;63(7):882-90.
11. Winkler B, Hufnagl K, Spittler A, et al. The role of Foxp3+ T cells in long term efficacy of prophylactic and therapeutic mucosal tolerance induction in mice. *Allergy*2006;61:173.
12. Roth-Walter F, Bergmayr C, Meitz S, et al. Janus-faced Acrolein prevents allergy but accelerates tumor growth by promoting immunoregulatory Foxp3+ cells: Mouse model for passive respiratory exposure. *Sci Rep*2017;7:45067.
13. Bianchini R, Roth-Walter F, Ohradanova-Repic A, et al. IgG4 drives M2a macrophages to a regulatory M2b-like phenotype:potential implication in immune tolerance. *Allergy*2019;74:483
14. Gieras A, Cejka P, Blatt K, et al. Mapping of conformational IgE epitopes with peptide-specific monoclonal antibodies reveals simultaneous binding of different IgE antibodies to a surface patch on the major birch pollen allergen, Bet v 1. *J Immunol*2011;186:5333.
15. Brier S, Le Mignon M, Jain K, et al. Chartacterization of epitope specifities of reference antibodies used for the quantification of the birch pollen allergen Bet v 1. *Allergy*2018;73:1032.
16. Jahn-Schmid B, Radakovics A, Lüttkopf D, et al. Bet v 1142-156 is the dominant T-cell epitope of the major birch pollen allergen and important for cross-reactivity with bet v 1-related food allergens. *J Allergy Clin Immunol* 2005;116:213.

**Supplementary Data – figure legends**

**Supplementary Figure S1: Geometry of Bet v 1a - RA complex and localization of (A) B-cell and (B) T-cell epitopes. (A)** Left diagram shows side chains of amino acids in both B-cell epitopes as sticks with carbons in slate blue (epitope 1: residues 29 - 58)^S14^ and salmon (epitope 2: residues 73 - 103)^S15^. Ligand RA is shown as stick with carbons in green. Red labels indicate all the epitope residues that are within 4 Å of the ligand (epitope 1: F31, K55, I57; epitope 2: Y82, Y84, I103). Right diagram displays partially transparent renderings of surface views of the geometries shown in the left diagram. **(B)** Left diagram shows side chains of amino acids in the T-cell epitope (residues 142 – 156)^S16^ as sticks with carbons in cyan. Ligands are shown as sticks with carbons in green. Red labels indicate the two epitope residues that are within 4 Å of the ligand (E142, L144). Right diagram displays partially transparent renderings of surface views of the geometries shown in the left diagram.

**Supplementary Figure S2: Bet v 1a- and Bet v 1d-specific IgE levels in sera from 12 BP-allergic individuals measured by ELISA. (A)** *apo*-Bet v 1 versus Bet v 1 loaded with RA, **(B)** *apo*-Bet v 1 versus Bet v 1 loaded with catechol, **(C)** *apo*-Bet v 1 versus Bet v 1 loaded with epinephrine; ** p < 0.01 by paired samples t-test; ns = non-significant.

**Supplementary Figure S3: Degradation of intact proteins upon endolysosomal degradation**. Time dependent proteolytic degradation of *apo-*Bet v 1a, *holo*-Bet v 1a, *apo-*Bet v 1d and *holo*-Bet v 1d was monitored by SDS-PAGE. No differences in protein stability of RA-bound or unloaded Bet v 1a were detected, while overall Bet v 1d was slightly more stable than Bet v 1a, independent of its loading state.

**Supplementary Figure S4: Therapeutic treatment scheme.** Sensitization of mice was performed by 3 intraperitoneal (i.p.) injections of 1 µg Bet v 1a adsorbed to aluminium hydroxide (Alum) at 14-day intervals. Therapeutic intranasal treatment (i.n.) was started 10 days after the last i.p. injection. Mice received 40 µg *apo*-Bet v 1a (*apo*-Bet v 1a group), 40 µg *apo*-Bet v 1a pre-incubated with RA (*holo*-Bet v 1a group), or as control RA alone (Co RA group) for 3 times in 6-days intervals. One week after the last intranasal treatment mice were challenged intraperitoneally with 30 µg Bet v 1a in PBS, anaphylactic reaction was recorded and sera were taken.

**Supplementary Figure S5: Percentage of CD4+CD25+ activated/regulatory T-cells** in splenocytes from Bet v 1-sensitized mice intranasally treated with *apo-*Bet v 1a, *holo*-Bet v 1a or RA alone.; mean+/- SEM; n.s. non-significant by one-way ANOVA followed by Tukey's multiple comparison test.
