# Supplementary figures and images for: Retinoic acid‐loading of the major birch pollen allergen Bet v 1 may improve specific allergen immunotherapy: In silico, in vitro and in vivo data in BALB/c mice
Source: Allergy. 2020 Apr 16;75(8):2073–7. doi: 10.1111/all.14259 (PMC7522679; doi:10.1111/all.14259)

**A**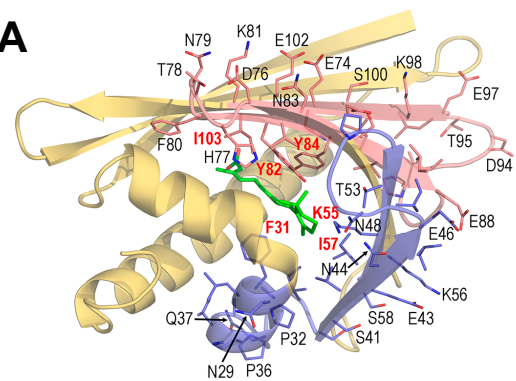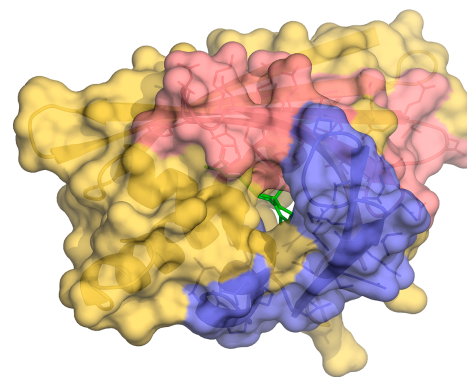**B**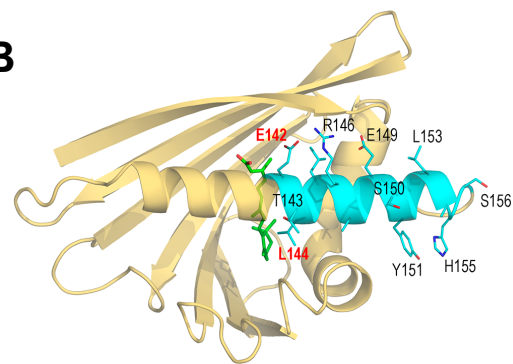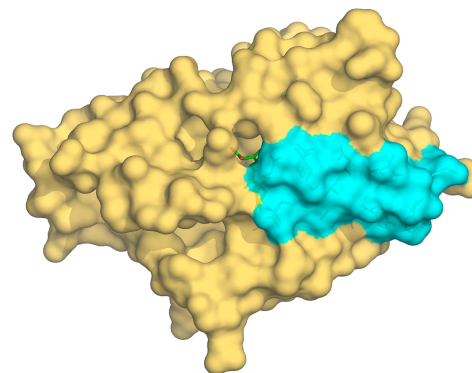

Supplement: Supplementary file 2 — Fig S1 [file ALL-75-2073-s002.pdf]

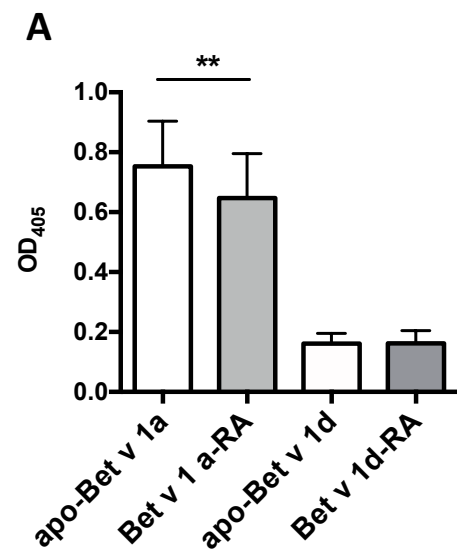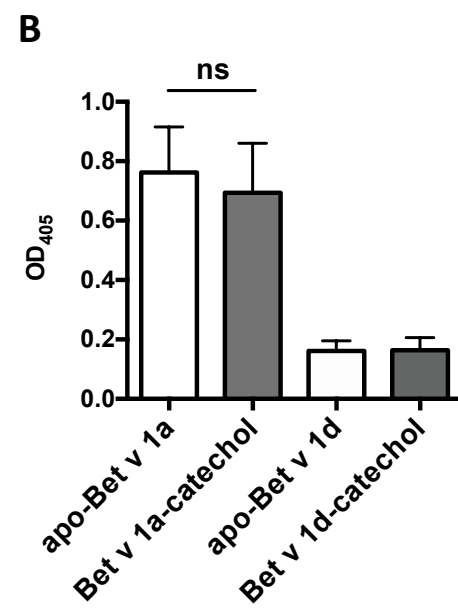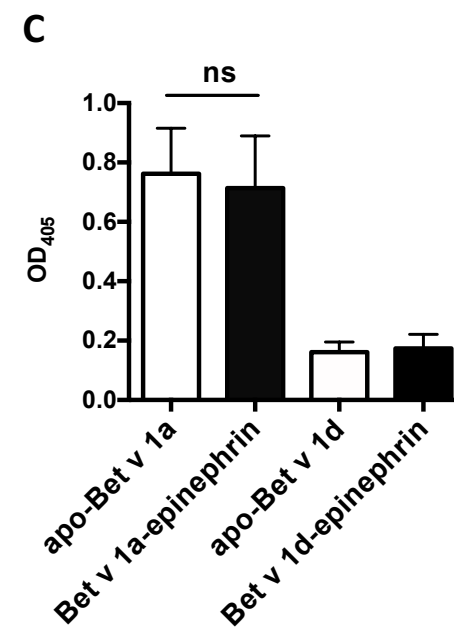

Supplement: Supplementary file 3 — Fig S2 [file ALL-75-2073-s003.pdf]

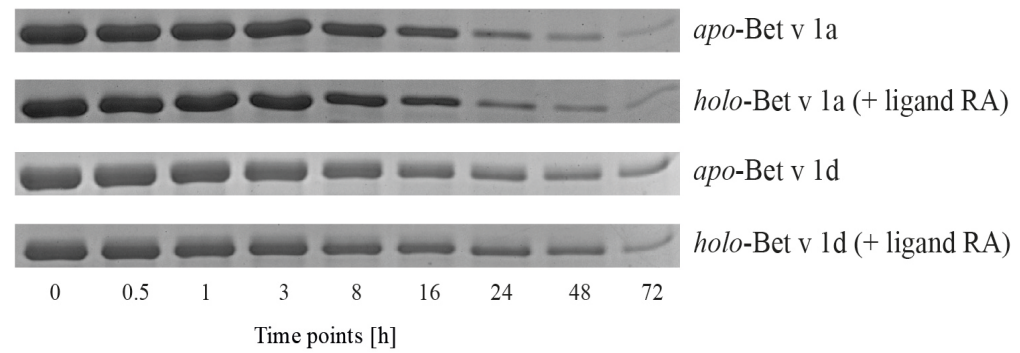

Supplement: Supplementary file 4 — Fig S3 [file ALL-75-2073-s004.pdf]

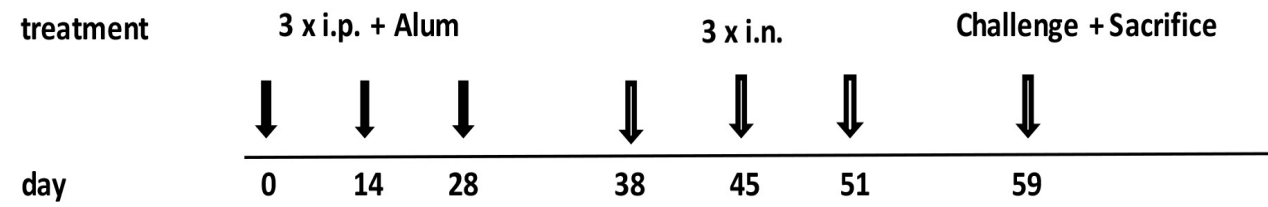

Supplement: Supplementary file 5 — Fig S4 [file ALL-75-2073-s005.pdf]

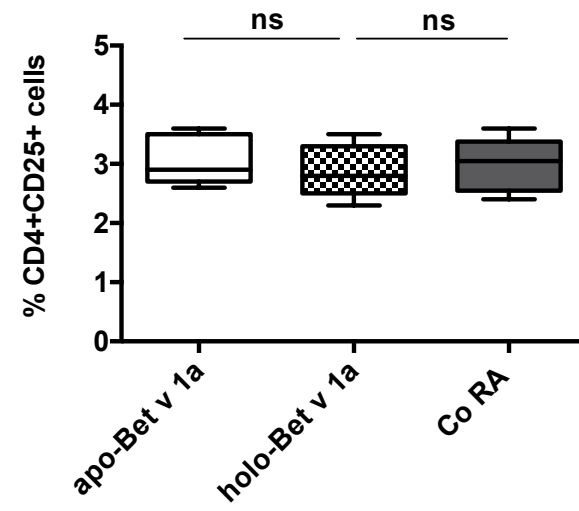

Supplement: Supplementary file 6 — Fig S5 [file ALL-75-2073-s006.pdf]
